# Predicting functional decline and survival in amyotrophic lateral sclerosis

**DOI:** 10.1371/journal.pone.0174925

**Published:** 2017-04-13

**Authors:** Mei-Lyn Ong, Pei Fang Tan, Joanna D. Holbrook

**Affiliations:** 1 Singapore Institute for Clinical Sciences (SICS), Agency of Science and Technology Research (A*STAR), Brenner Centre for Molecular Medicine, 30 Medical Drive, Singapore, Singapore; 2 NIHR Biomedical Research Centre, University of Southampton, Southampton General Hospital, Tremona Road, Southampton, United Kingdom; University of Rome La Sapienza, ITALY

## Abstract

**Background:**

Better predictors of amyotrophic lateral sclerosis disease course could enable smaller and more targeted clinical trials. Partially to address this aim, the Prize for Life foundation collected de-identified records from amyotrophic lateral sclerosis sufferers who participated in clinical trials of investigational drugs and made them available to researchers in the PRO-ACT database.

**Methods:**

In this study, time series data from PRO-ACT subjects were fitted to exponential models. Binary classes for decline in the total score of amyotrophic lateral sclerosis functional rating scale revised (ALSFRS-R) (fast/slow progression) and survival (high/low death risk) were derived. Data was segregated into training and test sets via cross validation. Learning algorithms were applied to the demographic, clinical and laboratory parameters in the training set to predict ALSFRS-R decline and the derived fast/slow progression and high/low death risk categories. The performance of predictive models was assessed by cross-validation in the test set using Receiver Operator Curves and root mean squared errors.

**Results:**

A model created using a boosting algorithm containing the decline in four parameters (weight, alkaline phosphatase, albumin and creatine kinase) post baseline, was able to predict functional decline class (fast or slow) with fair accuracy (AUC = 0.82). However similar approaches to build a predictive model for decline class by baseline subject characteristics were not successful. In contrast, baseline values of total bilirubin, gamma glutamyltransferase, urine specific gravity and ALSFRS-R item score—climbing stairs were sufficient to predict survival class.

**Conclusions:**

Using combinations of small numbers of variables it was possible to predict classes of functional decline and survival across the 1–2 year timeframe available in PRO-ACT. These findings may have utility for design of future ALS clinical trials.

## Introduction

Amyotrophic Lateral Sclerosis (ALS) is a progressive neurodegenerative disorder characterized by progressive degeneration of the upper and lower motor neurons throughout the central nervous system, and is associated with severe neurologic morbidity and death. The majority of patients die within 3–5 years following onset [1] mainly from the complications of respiratory failure. However, the clinical course of the disease is highly variable suggesting that multiple factors contribute to the pathogenesis of ALS with duration of the disease ranging from several months to more than 20 years [2]. Numerous therapies and experimental agents have been tested in ALS and have failed. Riluzole is the only licensed medicine that affects the ALS disease course and has only a modest effect on survival [3]. Thus, there remains a significant unmet medical need in ALS for therapies to slow progression of functional decline and improve survival.

Existing measures of assessment of ALS progression have limitations in terms of sensitivity and as a result require long trials with large sample sizes. There is a need to improve measures of disease progression and overall clinical study design. Better understanding, modeling and predicting progression of the disease would help design more efficient clinical trials of a shorter duration and/or with smaller sample sizes. For example, the study population could be enriched with faster progressing patients. Also, stratification based on predictive baseline values could be carried out to ensure balance in treatment and placebo arms or identify subsets of patients with a stronger efficacy signal and hence greater potential benefit. Furthermore, a robust model of ALS could potentially serve as a control group when the use of placebo may not be appropriate or feasible, or could allow a smaller control arm if used in combination.

In an effort to improve development of medicines for this devastating disease, the Prize for Life Foundation (http://www.prize4life.org/) have pooled de-identified clinical records data for ALS sufferers enrolled in late-stage clinical trials. This database is named The Pooled Open Access Clinical Trials Database (PRO-ACT) (www.ALSDatabase.org)[4, 5]. It includes data on all variables such as biomarkers that were collected in the individual studies. In the DREAM-Phil Bowen ALS Prediction Prize4Life challenge, PRO-ACT data pertaining to subjects in the first 3 months of their enrollment was used to predict decline in ALS-FRS over the next 9 months with accuracies exceeding chance and clinicians’ predictions and achieving root mean squared errors of ~54% [6]. Our study is also based on the PRO-ACT database. In our work, we attempted to build models to better predict decline in the gold standard ALSFRS-R score using parameters at baseline only. We also attempted to predict survival.

## Methods

### Dataset

Data used in the preparation of this article were obtained from the Pooled Resource Open-Access ALS Clinical Trials (PRO-ACT) Database. Data was extracted from the PRO-ACT database on 14 March 2014. Additionally, the Adverse Effect form was downloaded on 30 January 2015. Riluzole data was updated and re-downloaded on 07 April 2015. Data cleaning was performed as detailed in supplemental methods. As a general rule across all forms, data records with missing time points, ambiguous entries and non-numeric values were removed. Similarly, if duplicated time points existed per unique subject, mean values across available variables were computed. Also, columns with synonymous headings and mutually exclusive data with regard to subjects were merged. No imputations were performed. However, any missing data from ALSFRS-R, SVC and FVC that could be derived from the total score, were back calculated. Due to the large data size, the Laboratory Data form was split into 13 broad data categories to ease data cleaning procedures. There were 8,635 unique subjects recorded in the PRO-ACT database including 1,568 subjects with ALSFRS-R data. We included all available subjects regardless of whether they were in the placebo or interventional arm. The cleaned dataset contained 113 unique variables, mean age was approximately 56 years old, 40% of the subjects were female, 64% had limb onset and 65% had taken Riluzole (Table 1). The survival outcome was calculated for 6,355 subjects in the analysis. For 2976 subjects this was the recorded death day and for 3379 subjects (for whom last day of death was not recorded) the last day of follow-up was used as a proxy (S1 Table). The characteristics of the 6,355 were similar to the 1,568 subset for which ALSFRS-R was available (Table 1). All useable data was included in the dataset of 113 unique variables. This included all data from biomarker assays included in the original trials. We selected data only on the basis of completeness we did not de-select for relevance. Although we do not know why some biomarkers were or were not included in the trails, it is likely there was *a priori* reason to believe they could be predictive of disease course and/or they were pragmatically easy to measure in clinic. For cross-validation, each dataset was split into 60–40% for training and testing purposes respectively via cross validation, and this procedure was repeated one hundred times.

**Table 1:** Characteristics of 1,568 and 6,355 subjects for whom ALSFRS-R and survival data respectively were available in PROACT.

Data	ALS-FRS-R (n = 1568)	Survival (n = 6355)
Study characteristics	Percent or mean (Standard Deviation)	Percent or mean (Standard Deviation)
Age (years)	56.2 (11.78)	58.0 (11.37)
Height (cm)	169 (9.94)	169 (9.84)
Weight, Baseline (kg)	75.6 (18.31)	73.9 (19.23)
% Female	40.1	41
% Caucasian	56.6	56.3
Site of Onset; % Limb, % Bulbar	64.1, 19.1	61.1, 19.2
Baseline ALSFRS-R Score	39.4 (5.28)	39.4 (5.26)
Time in study (days)	314.9 (109.6)	309 (171)
% Riluzole	65.3	62.1
Number of subjects for whom day of death was recorded		2976 (47%)

### Data modelling

Time varied data for each subject was fitted to linear, exponential, harmonic and Weibull models. Parameter estimates for each model were obtained using simulated annealing (R “likelihood package” V1.7 https://cran.r-project.org/web/packages/likelihood/index.html). The model estimated baseline value at time point 0 was denoted as *A* and for the ALSFRS-R variable an upper bound of 53 was imposed. The rate of disease progression was denoted as *k* and restricted to range between -5 and 5 (following preliminary analyses where *k* was always estimated between 0–1) to speed up the optimization process. For the Weibull model, the shape parameter *b* was restricted to 0.0001 to 5 and when it approached the boundaries the model was collapsed to a 2-parameter exponential model. To assess the relationship between decline class and survival, cox regression survival analysis was performed in a univariate fashion.

### Model comparison

When comparing models via Akaike information coefficient accounting for small sample sizes (AICc) and root mean squared error (RMSE), we arranged their respective scores in ascending order (i.e. the smaller the values, the better the fit) and obtained the best, 2^nd^, 3^rd^ and 4^th^ ranked models. For AICc, we considered the absolute difference between the top and 2^nd^ best models to be insignificant if it is smaller than two [7]. If this criterion was true, we considered the 2^nd^ best model to be as good as the top model. Likewise for RMSE, we considered the percentage difference between the top and 2^nd^ best models to be insignificant if it is smaller than 10% of the top model [8]. If this criterion was true, we considered the 2^nd^ model to be as good as the top model.

### Machine learning algorithms

For each analysis, four algorithms: RUS/Ada Boost, Naïve Bayes, Decision Tree and Random Forest were run. The algorithms were chosen as capable of handling categorical outcomes and missing data in the variables. From the Boost suite of algorithms, RUS Boost was used for predicting fast and slow progressing patients as it is able to tolerate class imbalances; there are more subjects in the slow progression than fast progression category. This was not a factor for the high-low death risk, survival prediction because subjects are distributed more evenly so adaptive (Ada) Boost Decision Tree was used as it is less susceptible to over-fitting. The best models from each algorithm were compared with each other. Those with the higher area from a receiver operating characteristic curve (ROC) curve and lower nRMSE were declared the best performing model. In most cases this was the Boost algorithms, excepting the case of individualized ALSFRS-R *k* from baseline, for which the Random Forests model was reported.

S1 Fig provides a schematic representation of the analyses conducted.

## Results

### Time-series data was fitted to exponential models and binary categories were derived from the outcome variables

As time points when data were collected are not consistent across trials in PRO-ACT it is necessary to fit time-series data to a model, to allow merging of data across trials. ALSFRS-R data was fitted to linear, exponential, harmonic and Weibull models. The exponential model was the best fit for the majority of the subjects ALSFRS-R (62% by AICc and 78% by RMSE) therefore exponential models were used throughout. Parameters of baseline i.e. at t = 0 (*A*) and rate (*k*) were estimated for all time-series variables.

**Fig 1:**
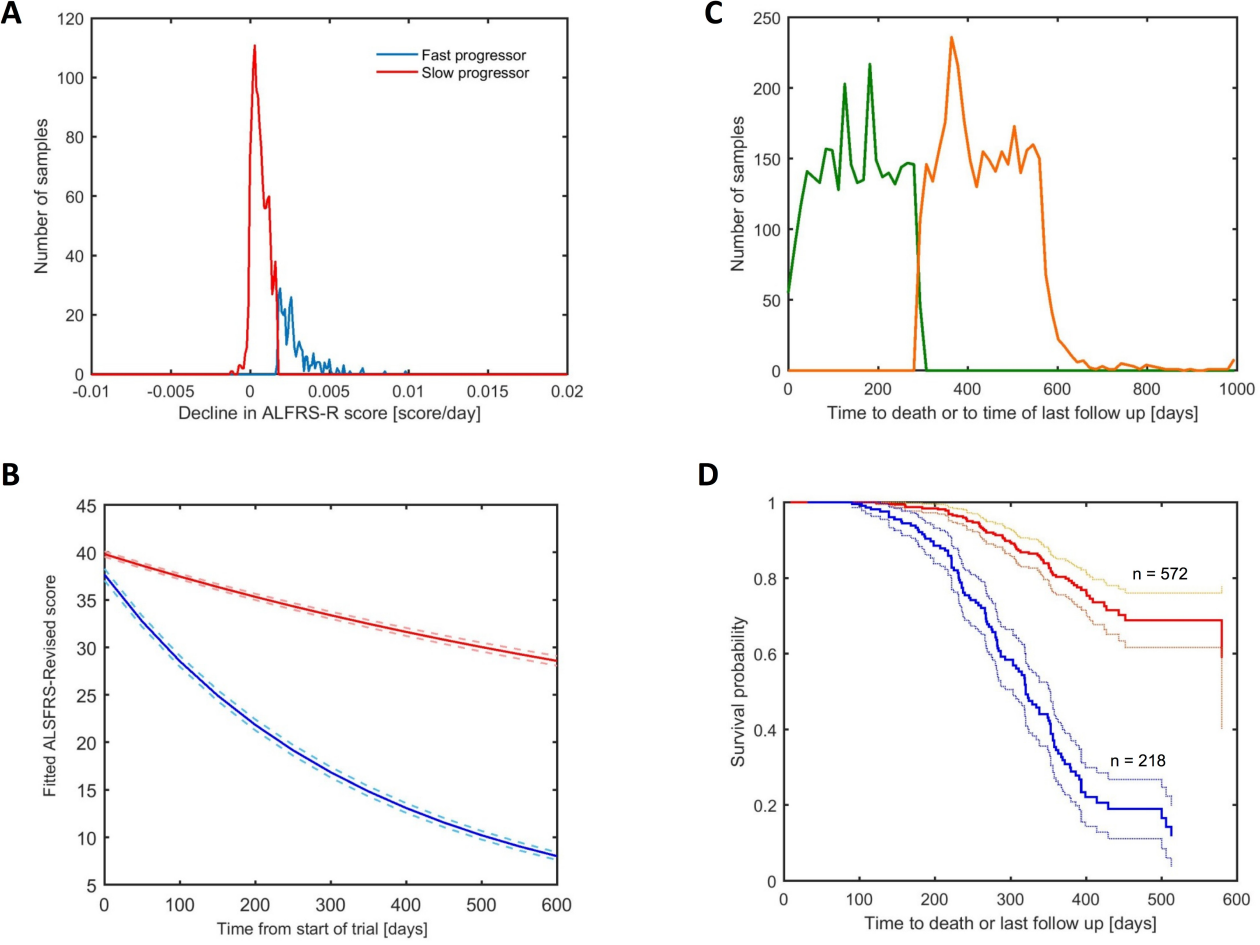
Subjects cluster into binary classes for progression and survival in PRO-ACT. (A) Frequency histogram of the fitted trajectory of ALSFRS-R score (*k*) in the exponential model. Lines are coloured via the k-means of the same parameter into decline class, fast progressing = blue, slow progressing = red. **(**B) Mean values of fitted trajectory of ALSFRS-R score (*k*) in the exponential model over a 6 month time period for for the k-means clustering derived decline classes, fast progressing = blue, slow progressing = red (as in 1A). Dotted lines represent 95% confidence bounds. (C) Frequency histogram of the time to death or time of last follow-up. Lines are coloured via subject classification into survival classes (as opposed to the progression classes in 1A, B and D) derived from k-mean clustering of the number of days from the start of the trail to day of death or last follow-up contact date, high death risk = green, low death risk = orange. (D) Kaplan-Meier plot of estimated survival probability by decline class (again derived from k-means clustering of the fitted trajectory of ALS-FRS-R score (*k*) in the exponential model, akin to 1A and 1B) using univariate Cox regression. The fast progressing group (blue n = 218) has an increased death risk compared to the slow progressing group (red n = 572) (hazard ratio = 4.21, p<2x10^-16.^)

To avoid modeling artifacts due to noise in the data, the 1,568 subjects with ALSFRS-R data were categorized into two groups using partitioning around medoids (PAM) and K-means clustering on their decline parameter (*k*). Both methods implied a binary classification was optimal. The k-means cluster categorisation was used for subsequent analyses since it showed a slightly higher median ALSFRS-R rate difference between the two groups (Fig 1A) than that obtained by PAM. They were denoted fast and slow progressing patients and there was an estimated median 20 point drop in ALSFRS-R score over a 6 month period (3.3 points /month) for the fast progressing compared to a 3 point drop (0.5 points / month) for the slow progressing patients (Fig 1B).

The survival time was derived by number of days from start of the trial until death, for the 2976 subjects for whom a death date was available. For the 3379 subjects for whom a death date was not available, the last follow-up contact date was used as a proxy for the date of death (S1 Table). This incorporates the obvious bias that number of days until death is underestimated in the latter group. In order to reduce the impact of the bias in our dataset (as well as reduce error due to model fitting), the survival outcome for the 6,355 subjects was subjected to unsupervised clustering by K-means and PAM. A binary outcome was optimal for data fit. The two clusters were termed high and low death risk. The threshold at which the two groups were defined was 291 days (~10 months) from the start of the trial (Fig 1C). As the trials within PRO-ACT on average lasted 309 days, more than 10 months we expect this categorisation to reduce error introduced by underestimating the time to death (by using the proxy of last follow-up day). However, an important limitation to this approach is that participants in studies shorter than 10 months, who do not have a death date recorded will be uniformly categorized as high risk. To investigate the effect of this limitation, the clustering was performed only on the 2976 subjects with an actual date of death. Again two clusters resulted with a separation of ~10 months, the median time to death in the low death risk group was 338 days (compared to the 433 days for the low death risk cluster in the full dataset) and the median time to death in the high death risk group was 142 days (compared to 150 days in the high death risk cluster in the full dataset) (S2 Table). The proportion of subjects classified as high risk was similar (and actually slightly lower) in the full dataset including subjects with last day of follow-up used as a proxy for date of death (47% of 6355) compared to the dataset consisting only of those subjects with actual death day recorded (49% of 2976)

Cox regression for survival using fast or slow progression as a predictor, unsurprisingly found that the fast progressing patients had an increased risk compared to the slow progressing patients (hazard ratio = 4.21, p<2x10^-16^) (Fig 1D). 790 subjects had both decline and survival classifications: 138 of the 218 fast progressing patients had high death risk (63%), whilst 324 of the 572 slow progressing patients (57%) had high death risk (Table 2). Mean baseline ALSFRS-R scores were ranked highest to lowest for the slow progressing and low death risk patients, slow progressing and high death risk patients, fast progressing and low death risk patients and fast progressing and high death risk patients (S3 Table).

**Table 2:** Distribution of the 790 subjects with both decline and survival classifications.

	Survival		
**Estimated decline class**		High death risk*	Low death risk
	Fast progressing patients	138 (17.5%)	80 (10.1%)
	Slow progressing patients	324 (41.0%)	248 (31.4%)

* includes subjects with last date of follow-up used as a proxy for date of death

### Trajectory of weight, alkaline phosphatase, albumin and creatine kinase was able to predict ALSFRS-R decline class with fair accuracy (AUC = 0.82)

**Fig 2:**
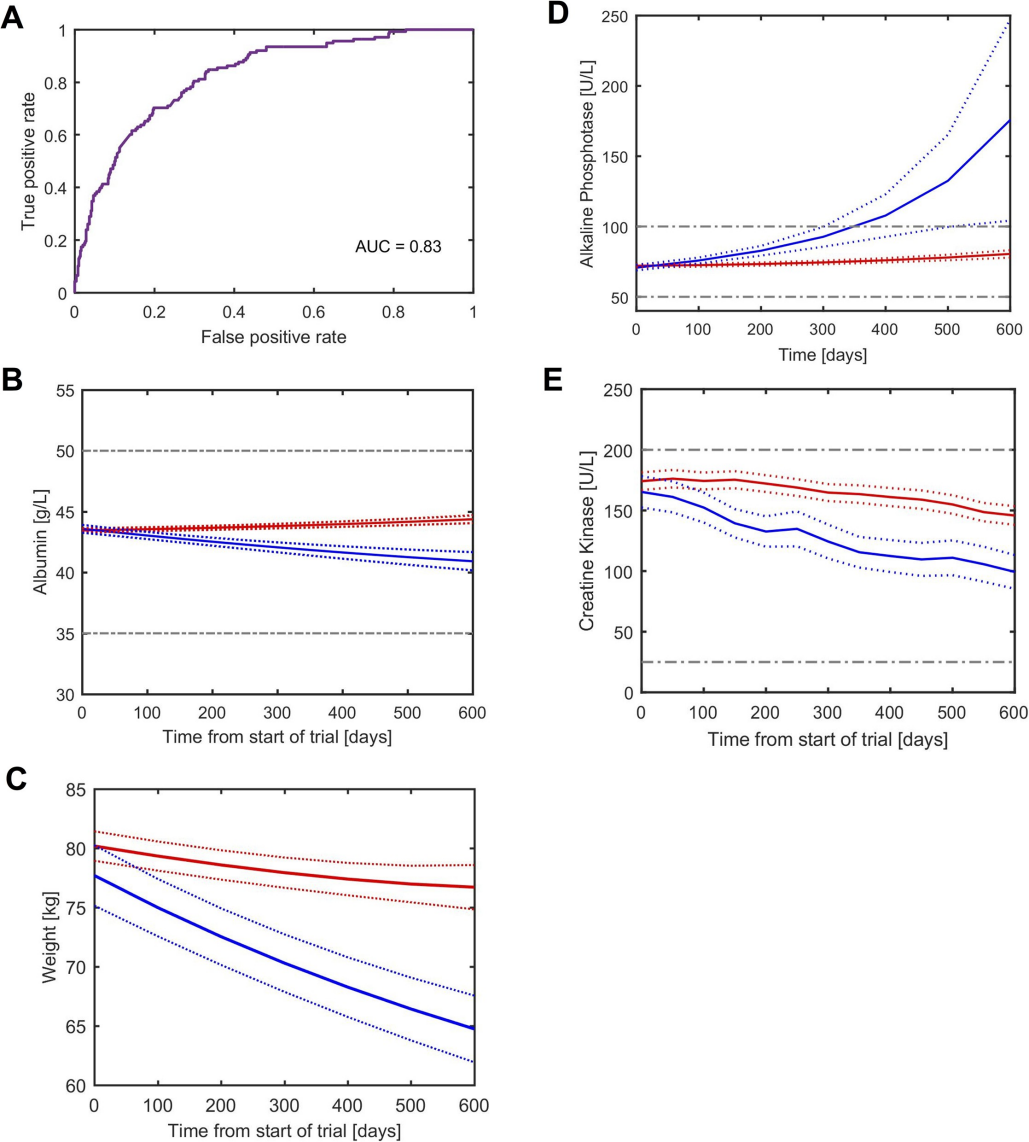
Decline class is predicted by trajectory of non-ALSFRS-R variables. (A) Prediction accuracy by receiver operating curve (ROC) for model-defined decline class using baseline (A) and trajectory (*k*) for all 113 variables excluding ALSFRS-R subscales, FVC and SVC in RUS-Boost model. (B-E) Estimated trajectory (*k*) for (B), albumin (C), weight (D), alkaline phosphatase (E), creatine kinase (y-axis) against time (x-axis) for model-defined fast-progressing (blue) and slow progressing patients (red). Dotted lines represent 95% confidence interval bounds. Gray dashed lines represent reference ranges for healthy adults.

Parameters describing all 113 unique variables (i.e. A and *k* for time-series data) excluding ALSFRS-R subscales, FVC and SVC measures were entered into machine learning models to attempt to predict decline class. The RUS-Boost model performed best and a cross-validation AUC of 0.82 was achieved (Fig 2A). The trajectories (*k*) of top four predictive variables were sufficient to achieve the same accuracy as the full model, they were (in order of importance): albumin, weight, alkaline phosphatase and creatine kinase. Albumin concentration decreased in the fast progressing patients and increased in the slow progressing patients (Fig 2B), weight declined more steeply (Fig 2C), alkaline phosphatase increased more sharply (Fig 2D) and creatine kinase decreased more sharply (Fig 2E) in the fast progressing patients compared to the slow progressing patients. All four trajectories were nominally significantly different (p<0.05) but baseline characteristics for the predictive variables between groups were nominally significantly different (p<0.05) only for creatine kinase, which was higher in the slow progressing patients compared to fast, at baseline (S4 Table).

### Baseline variables did not accurately predict ALSFRS-R decline class but were able to fairly accurately predict individual slope of decline with nRMSE = 15%

**Fig 3:**
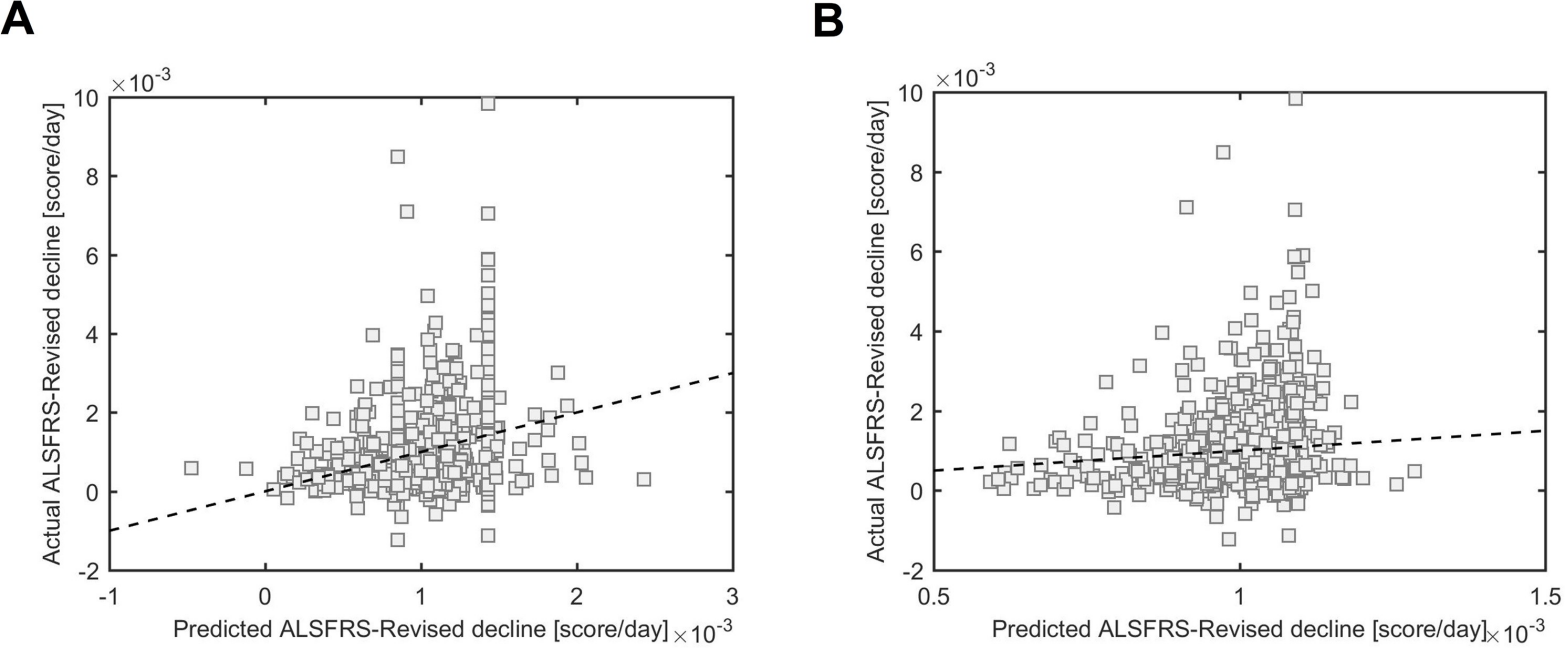
Fitted decline rate (*k*) is predicted from baseline variables. Predicted values are on the x-axis and actual values on the y-axis. Random forests models (A) or Ada boosting models using regression trees (B) were used.

We attempted to use only the baseline variables (i.e. all 113 variables in the dataset at beginning of the study (t = 0). For time-series data this means the fitted A parameter representing estimated value at t = 0) to predict decline class. We excluded ALSFRS-R subscales. However the best model (using RUS Boost) achieved only very poor prediction accuracy (74% of slow progressing patients were predicted as such but only 39% of actual fast progressing patient were predicted as such) (Table 3). Surprisingly, when we performed random forest and Ada boosting using regression trees on all variables at baseline (excluding ALSFRS-R subscales) to predict individual slope of ALSFRS-R decline (*k*) as opposed to decline class we were able to achieve reasonable predictive power. Both models performed similarly and predicted ALSFRS-R decline with nRMSE = 15% (Fig 3). After variable selection, eight variables were sufficient to recapitulate the prediction accuracy of the random forests model and nine variables were sufficient in the Ada Boost model (Table 4). Weight, pulse and blood pressure were common to both models.

**Table 3:** Prediction accuracy of class of decline using baseline variables (value at t = 0) only.

	Predicted decline class accuracy (%)		
**Actual decline class**		Slow progressor	Fast progressor
	Slow progressor	74	26
	Fast progressor	61	39

**Table 4:** Variables predictive of ALSFRS-R decline rates ranked by importance score for Random Forest and LS boosting models.

Random Forest	Ada boost using decision trees
1. Weight	1. Bilirubin total
2. Kidney Creatinine	2. Lactate Dehydrogenase
3. Pulse	3. Standing pulse
4. ALT (SGPT)	4. Standing BP systolic
5. Site of onset	5. Standing BP Diastolic
6. Standing BP (Diastolic)	6. Weight
7. Chloride	7. Gamma glutamyltransferase
8. FVC Percent Normal	8. Protein
	9. BMI

### Survival class was fairly accurately predicted using the trajectories of five parameters (AUC = 0.83)

**Fig 4:**
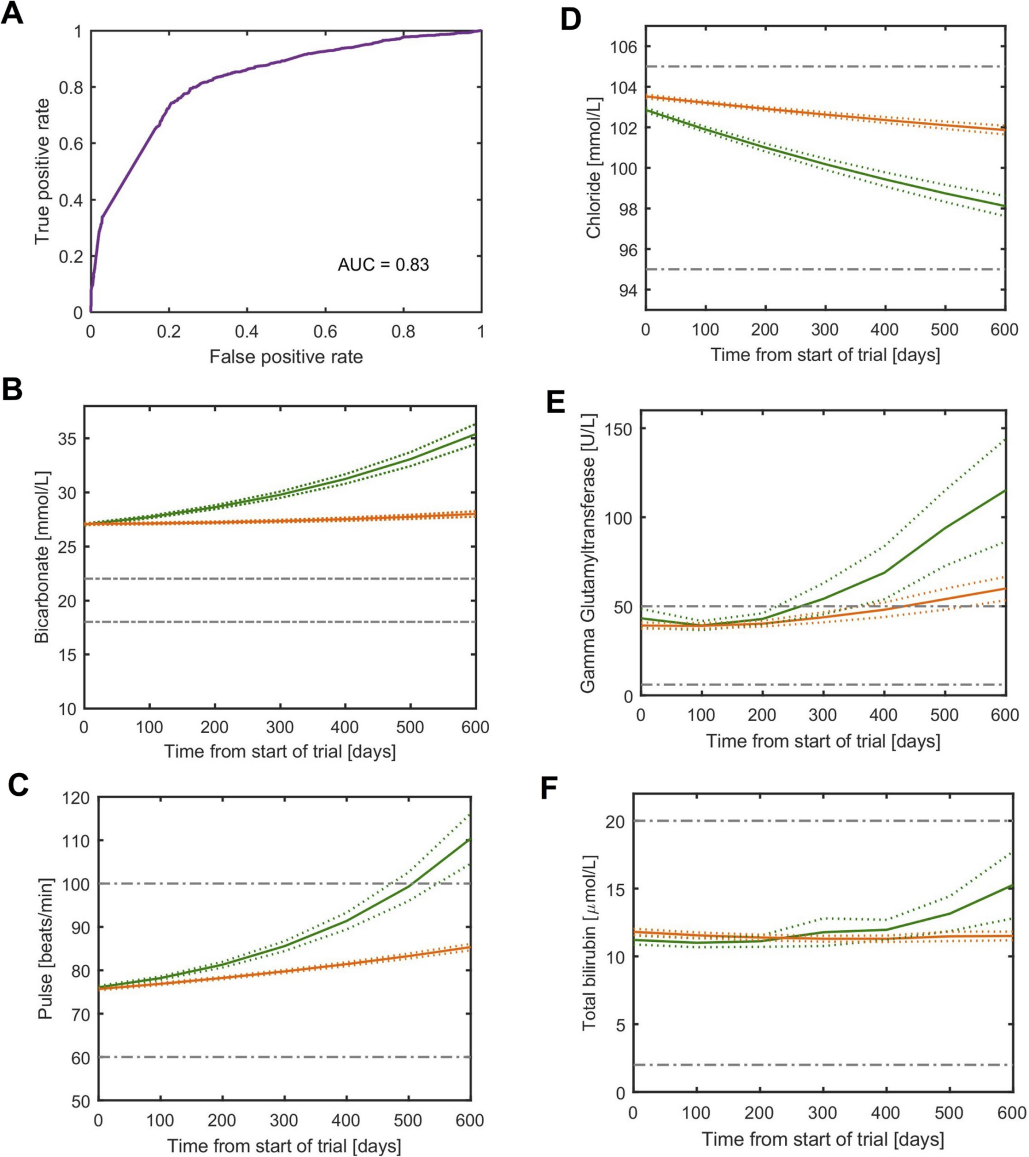
Survival class is predicted by trajectory of five variables. (A) Prediction accuracy by receiver operating curve (ROC) for model-defined survival class (includes subjects with last date of follow-up used as a proxy for date of death) using baseline (*A*) and trajectory (*k)* for all variables (except ALSRS-R subscales) in Ada-Boost model. (B-F), Estimated trajectory (*k*) for (B), bicarbonate C, Pulse D, chloride E, gamma glutamyltransferase F, total bilirubin (y-axis) against time (x-axis) for model-defined high death risk group (green) and low death risk group (orange). Dotted lines represent 95% CI bounds. Gray dashed lines represent reference ranges for healthy adults.

All variables (excepting ALSFRS-R subscales) were entered into the machine learning models to attempt to predict survival class. Ada Boost correctly predicted 77% of high death risk subjects and 76% of low death risk subjects as such (Table 5). The AUC was 0.83 (Fig 4A). Variable selection resulted in five parameters, whose trajectories (*k*) were sufficient to achieve the full model’s classification accuracy: bicarbonate, total bilirubin, gamma glutamyltransferase, chloride and pulse. Subjects in the high death risk group, tended to have a higher rate of bicarbonate concentration increase (Fig 4B) and pulse increase (Fig 4C) and higher rate of chloride decrease (Fig 4D), gamma glutamyltransferase increase (Fig 4E) and total bilirubin increase (Fig 4F) compared to the low death risk group. Interestingly chloride, bicarbonate and pulse were significantly different between the two groups at baseline (Fig 4D and S5 Table).

**Table 5:** Prediction accuracy of survival class using baseline (*A*) and trajectory (*k*) for all variables (except the ALSRS-R subscales) in Ada-Boost model.

	Predicted survival class accuracy (%)		
**Actual survival class**		High death risk *	Low death risk
	High death risk	77.3	22.7
	Low death risk	23.9	76.1

* includes subjects with last date of follow-up used as a proxy for date of death

### Survival class was also fairly accurately (AUC = 0.8) predicted using four variables measured at baseline

**Fig 5:**
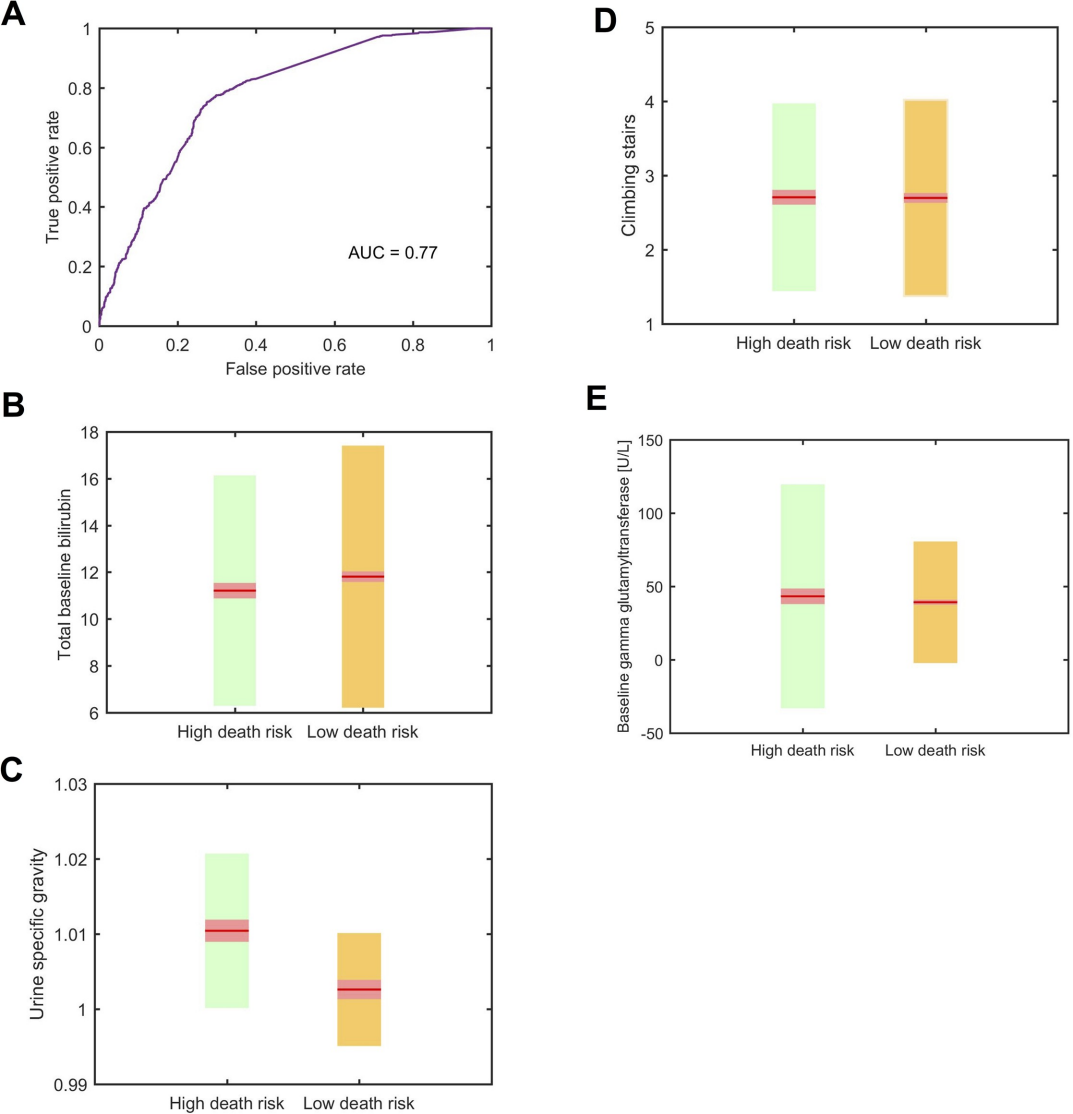
Survival class is predicted from baseline variables. (A) Prediction accuracy by receiver operating curve (ROC) for model-defined survival class (includes subjects with last date of follow-up used as a proxy for date of death) using baseline for all variables in Ada-Boost model. (B-E) Fitted baseline values showing mean, 95% confidence interval and one standard deviation for (B) bilirubin, (C) urine specific gravity, (D) climbing stairs, (E) gamma glutamyltransferase (y-axis) for model-defined high death risk group (green) and low death risk group (orange).

When all the variables available to the machine learning algorithms were restricted to fitted values at baseline only (*A*), the Ada Boost algorithm predicted survival class with only slightly reduced accuracy (72% and 75% of high death risk and low death risk subjects were correctly classified, respectively—Table 6). The AUC was calculated to be 0.77 (Fig 5A). After variable selection, the baseline values of total bilirubin, gamma glutamyltransferase, urine specific gravity and ALSFRS-R climbing stairs item score together resulted accuracy similar to the full model. Subjects in the high death risk group tended to have lower total bilirubin at baseline (Fig 5B) and higher values for each of the following parameters: urine specific gravity (Fig 5C), climbing stairs (Fig 5D) and gamma glutamyltransferase (Fig 5E) values at baseline, compared to subjects in the low death risk group.

**Table 6:** Prediction accuracy of survival class using baseline (*A*) for all variables in Ada-Boost model.

	Predicted survival class accuracy (%)		
**Actual survival class**		High death risk *	Low death risk
	High death risk	71.9	28.1
	Low death risk	25.4	74.6

* includes subjects with last date of follow-up used as a proxy for date of death

S1 Fig provides a schematic representation of all the analyses conducted.

## Discussion

We found that patients in the PRO-ACT database can be separated into two groups based on progression of the disease over time (Fig 1A and 1B) as estimated from their fitted ALSFRS-R decline slopes. 1237 and 331 subjects were classified as slow and fast progressing patients, respectively. It may be that this grouping can inform patient stratification and clinical trial recruitment and data analysis. Other authors also found evidence for subgroups within the PRO-ACT subjects. Gomeni et al (2014) used Weibull models to describe ALSFRS-R progression in only the placebo arms of PRO-ACT and in very similar results to those presented here, found two overlapping categories of slow and fast progressing patients although at roughly equal subject proportions [9]. The authors suggested that trials enriched for fast progressing patients may be more successful at showing therapeutic benefit. In this study, we found that fast progressing patients were less prevalent than slow progressing patients (21% compared to 79%).

Gomeni et al (2014) also showed that the fast/slow progression category could be predicted from ALSFRS-R data from 2–4 weeks after trial start but not from only baseline characteristics (i.e. data at t = 0) only [9]. In this study, we were also unable to build a predictive model for decline category from baseline data only (Table 3) but we were able to show that the category can be predicted by decline in non-ALSFRS-R subscale variables, i.e. *k* for weight, alkaline phosphatase, albumin and creatine kinase (Fig 2). As might be expected slow progressing patients have slow weight decrease whilst fast progressing patients have fast weight decrease as a reflection of muscle atrophy in ALS. Similarly, slow progressing patients had a stable or slowly declining creatine kinase levels whilst fast progressing patients had a fast decline. Rafiq et al. 2016 [10] showed that higher creatine kinase levels were associated with better survival, the observation which is in line with the findings of the current study. It was suggested that these changes might reflect compensatory upregulation of creatine kinase as an additional pathway to provide energy in response to hypermetabolic state in ALS [10]. Alkaline phosphatase levels were stable in slow progressing patients while fast progressing patients tend to have sharply increasing alkaline phosphatase. This is consistent with other studies that have found increased alkaline phosphatase in a subset of ALS patients and those with related disorders [11]. Lastly, slow progressing patients tend to have flat or increasing albumin whilst fast progressing patients have decreasing albumin. Better survival was previously linked with higher levels of albumin [12].

Interestingly, we were able to predict individualized ALSFRS-R decline from baseline variables (Fig 3). Our model achieves similar accuracy to that reported by Kuffner et al (2015) [6] who used data from 3 months post baseline including ALS-FRS total score and its subscales to predict ALS-FRS decline (slope) in the next 9 months. Many predictive variables were shared between the models presented here and by Kuffner et al including creatinine, weight, pulse and blood pressure. Weight and creatinine were also highlighted as predictive variables in multivariate models for survival by Lunetta et al (2015) [13]. There are several possible explanations for the discrepancy in our success in predicting individual decline based on baseline parameters compared to progression category prediction. It may be that prediction accuracy for individual scores reflects over fitting to small inter-individual differences introduced by inaccurate model fit to the data. On the other hand, grouping patients into binary progression categories, as we have done, may remove too much individual heterogeneity to then achieve accurate prediction from baseline.

In this study we also found that survival could be classified into two categories of subjects (Fig 1C). It is interesting that the functional decline category based on ALSFRS-R total score was strongly associated with survival (Fig 1D) but is not a strong predictor (Table 2). The imperfect association between ALSFRS-R decline and survival has been shown by others e.g. [14–16]. We attempted to improve on survival prediction using other variables in the PRO-ACT database. We were able to predict survival class with some accuracy using a combination of concurrent measures (Fig 4, Table 5). The changes of the parameters having a predictive value in this model, such as more rapidly increasing bicarbonate and decreasing chloride in the high death risk group compared to the low risk group, are likely to reflect chronic respiratory acidosis with compensatory increase in bicarbonate and increased chloride excretion in patients with more severe respiratory disturbances, and respiratory failure is a main cause of death in ALS patients [1]. In our analysis, the high death risk subgroup had a slightly lower total bilirubin levels at baseline compared to the low death risk group (Fig 5B). However, in the high death risk subgroup total bilirubin increased over time (while still remaining within normal limits, Fig 4F) with the low death risk group following the opposite direction with much smaller overall change of total bilirubin. Although biological significance of the findings is not clear; decreased bilirubin was shown to be associated with longer duration of ALS and was hypothesized to reflect decompensation of endogenous antioxidant systems [17] with hypermetabolic state as a part of the disease being the alternative explanation [18]. We were also able to predict survival class at baseline with reasonable accuracy using only four variables: total bilirubin, gamma glutamyltransferase, urine specific gravity and climbing stairs from the ALSFRS-R subscales (Table 6, Fig 5). The differences in the prognostic parameters for the two outcomes of functional decline and survival highlight the differences in disease progression. The underlying mechanisms for the changes in individual laboratory parameters are not completely understood, as these could be linked to ALS pathophysiology, represent compensatory changes or possibly reflect effects of the disease on the body systems. The predictive power of bilibrubin and gamma-glutamayltransferase levels implicate liver metabolism in ALS disease course. Energy and lipid metabolism have been identified as independent prognostic factors for survival in ALS [19, 20][21].

A major limitation of this study was that it was conducted solely based on the PRO-ACT database and although care was taken to evaluate the predictive models by cross-validation, over-fitting to the confounder structure remains a possibility without an independent replication set. An obvious source of confounding in PRO-ACT is the data from different clinical studies of different duration. There is likely to be differences in survival between studies conducted at different times as there has been improvement in standard of care management for example in quality of respiratory support. Also differential outcomes might be expected for the control and intervention study arms, although none of the studies included showed significant efficacy. Studies are pooled and anonymised, however it is possible to derive study as a surrogate variable based on similarity of data structure between subjects (although its impossible to identify the actual studies). This surrogate variable along with control or intervention assignment, was included in the predictive models but neither was ever a selected variable in the reduced models, suggesting study ID and intervention arm did not represent a strong source of confounding.

An additional source of error in our study was that actual death day was not known for 53% of patients and last day of known survival (i.e. last day of follow-up) was used as a proxy for the date of death. To minimize this source of error we produced binary categories for the survival variables. However, this is likely to reduce rather than eliminate the problem. If there are studies of less than 10 months duration in the database. All subjects in the short study would be classified as high risk. The facts that the derived study variable is not predictive in our models and that clustering performed on only those subjects for whom last day of death is available resulted in very similar categories with no decrease in the proportion of subjects in the high death risk group, suggest that there is some validity in our approach but caution is very much warranted when interpreting the results. We look forward to replication in other datasets and the development of statistical tools to gain insights from datasets, such as this, with high proportions of missing data.

Finally, the identified predictive variables should not be interpreted as necessarily causally implicated in ALS progression or survival, there was a complex inter-dependencies in the predictive variables and the variable identified as important in the predictive models may be proxies for other variables not selected.

In this study, survival class was more accurately predicted by baseline parameters than functional decline class. One of the possible explanations for this is that functional decline measured by ALSFRS-R is a more subjective outcome. Whereas these limitations could be addressed by ensuring a consistent and standardized approach in ALSFRS-R administration, there is also a need for novel, more sensitive outcome measures. Further investigation is warranted to determine if there is a combination of parameters that can provide a more robust measure of disease course. As baseline characteristics were not predictive of disease course in this study, we suggest that the availability of biomarker data during disease progression is important in the design of future studies. This study suggests that parameters predictive of overall survival may contribute to reliable predictions of individual patient’s progression trajectory, which in turn could possibly help to design better studies to accelerate development of new treatments for ALS.

## Supporting information

S1 Fig Schematic of approach and summary of results from combinations of variable sets. Variables available to the learning algorithms included fitted baselines values (*A)* and trajectories *(k)*. Three different outcomes were predicted: (i) model-defined binary survival classes (includes subjects with last date of follow-up used as a proxy for date of death), (ii) model-defined binary decline classes and (iii) fitted continuous rate of ALSFRS-R progression. Five analyses were performed and results are presented in the top right of the diagram. Performance of the machine learning models are denoted by area under the curve (AUC) or normalised root mean squared errors (nRMSE). Failed indicates inability to produce a model that performed better than chance.
(TIF)

S1 Table Number of actual deaths in the high and low death risk categories. (PDF)

S2 Table Median time to death (days) for clustering performed on only the 2976 subjects with actual death data. (PDF)

S3 Table Baseline ALSFRS-R total score mean and standard deviations of the 790 subjects with both decline and survival classifications. (PDF)

S4 Table Baseline characteristics of variables associated with decline. (PDF)

S5 Table Baseline characteristics of variables associated with death risk. (PDF)
