# Supplementary material for: Predicting functional decline and survival in amyotrophic lateral sclerosis
Source: PLoS One. 2017 Apr 13;12(4):e0174925. doi: 10.1371/journal.pone.0174925 (PMC5390993; doi:10.1371/journal.pone.0174925)
Supplement: S1 Table — (PDF) [file pone.0174925.s002.pdf]

Supplementary Table 1

Table S1: Number of actual deaths in the high and low death risk categories

Survival class	Subjects with recorded death day	Subjects for whom last day of follow-up was used as a proxy
High death risk	1630	1356
Low death risk	1346	2023
Total	2976	3379
