# Supplementary material for: Predicting functional decline and survival in amyotrophic lateral sclerosis
Source: PLoS One. 2017 Apr 13;12(4):e0174925. doi: 10.1371/journal.pone.0174925 (PMC5390993; doi:10.1371/journal.pone.0174925)
Supplement: S2 Table — (PDF) [file pone.0174925.s003.pdf]

# Supplementary Table 2

**Table S2: Median time to death (days) for clustering performed on only the 2976 subjects with actual death data**

| | Low Death Risk | High Death Risk |
|---|---|---|
| **Subjects with actual death data, n=2976** | **Median days to death** | **Median days to death** |
| | 338 days, n=1504 subjects | 142 days, n=1472 subjects |
