# Supplementary material for: Predicting functional decline and survival in amyotrophic lateral sclerosis
Source: PLoS One. 2017 Apr 13;12(4):e0174925. doi: 10.1371/journal.pone.0174925 (PMC5390993; doi:10.1371/journal.pone.0174925)
Supplement: S3 Table — (PDF) [file pone.0174925.s004.pdf]

Supplementary Table 3

Table S3: Baseline ALSFRS-R total score mean and standard deviations of the 790 subjects with both decline and survival classifications

		Survival	
		High death risk	Low death risk
**Estimated decline class**	Fast progressor	36.9 (5.1)	37.5 (6.0)
	Slow progressor	38.7 (5.2)	39.7 (4.8)
