# Supplementary material for: Predicting functional decline and survival in amyotrophic lateral sclerosis
Source: PLoS One. 2017 Apr 13;12(4):e0174925. doi: 10.1371/journal.pone.0174925 (PMC5390993; doi:10.1371/journal.pone.0174925)
Supplement: S4 Table — (PDF) [file pone.0174925.s005.pdf]

Supplementary Table 4

Table S4: Baseline characteristics of variables associated with decline

	Slow Progressor	Fast Progressor	Logistic regression
**Baseline variables**	**Mean (Standard Deviation)**	**Mean (Standard Deviation)**	**pvalue**
**Weight (kg)**	80.2 (22.3)	77.7 (23.6)	ns
**Albumin (g/L)**	43.5 (3.2)	43.6 (3.0)	ns
**Alkaline Phosphatase (U/L)**	72.1 (21.2)	70.7 (18.0)	ns
**Creatine Kinase (U/L)**	174.1 (79.8)	165.3 (75.8)	0.039
