# Supplementary material for: Predicting functional decline and survival in amyotrophic lateral sclerosis
Source: PLoS One. 2017 Apr 13;12(4):e0174925. doi: 10.1371/journal.pone.0174925 (PMC5390993; doi:10.1371/journal.pone.0174925)
Supplement: S5 Table — (PDF) [file pone.0174925.s006.pdf]

Supplementary Table 5

Table S5: Baseline characteristics of variables associated with death risk

	Low Death Risk	High Death Risk	Kaplan-Meier analysis of survival	
**Baseline variables**	**Mean (Standard Deviation)**	**Mean (Standard Deviation)**	**% increase in hazard**	**pvalue**
**Bicarbonate (mmol/L)**	27.1 (2.7)	27.0 (2.8)	-2.4	0.038
**Pulse (beat/min)**	75.7 (9.1)	76.1 (9.9)	1.2	8.94E-05
**Chloride (mmol/L)**	103.0 (3.0)	102.9 (3.0)	-10.4	<1E-6
**Gamma Glutamyltransferase (U/L)**	39.2 (41.5)	43.2 (76.3)	0.08	ns
**Bilirubin Total (umol/L)**	11.8 (5.6)	11.2 (4.9)	0.16	ns
